# Epidemiology of hip and groin injuries in Swedish male first football league

**DOI:** 10.1007/s00167-019-05470-x

**Published:** 2019-03-20

**Authors:** Filip Lundgårdh, Kjell Svensson, Marie Alricsson

**Affiliations:** 1 Department of Health Sciences, Swedish Winter Sport Research Centre, Mid Sweden University, Östersund, Sweden; 2 Department of Molecular Medicine and Surgery, Stockholm Sports Trauma Research Centre, Karolinska Institutet, Stockholm, Sweden; 3 Department of Sports Science, Linnaeus University, 391 82 Kalmar, Sweden

**Keywords:** Burden, Incidence, Injury rate, Professional, Risk factors, Soccer

## Abstract

**Purpose:**

This study aimed to investigate the incidence, pattern, and burden of hip/groin injuries in Swedish professional male football players over five consecutive seasons.

**Methods:**

Injury history from 16 football teams in the Swedish male first football league was evaluated during five consecutive seasons. The team’s medical staff recorded team exposure and time-loss injuries prospectively between 2012 and 2016.

**Results:**

In total, 467 time-loss injuries located in the hip/groin area were recorded among 1,687 professional male football players, with an overall incidence and burden of 0.82/1,000 h and 15.6/1,000 h, respectively. There appeared to be an increased risk of hip/groin injuries during the last two seasons (2015–2016); however, the difference was not statistically significant (n.s). Recurrent injury rate was relatively low (14%), and overuse injuries accounted for the majority of injuries and absence days. Muscle injuries were the main injury type, while kicking and sprinting/running were the primary causes of injury. Goalkeepers had the lowest percentage of injuries and absence days.

**Conclusion:**

Hip/groin injuries are a substantial problem in football, but does not seem to be an increasing phenomenon in the Swedish male first football league. Index and overuse injuries accounted for the majority of injuries and absence days. Thus, the focus should be on preventing hip/groin injuries to lower the injury rate. These new findings should be taken into consideration when designing and implementing preventive training interventions.

**Level of evidence:**

II.

## Introduction

The hip/groin is one of the most injury prone areas in football [7]. It constitutes approximately 11–17% of all injuries in professional football [37], with frequencies as high as 19% [32].

However, the hip/groin is, difficult to diagnose and treat due to the complexity of the region [38] and the lack of consensus statements [36]. As an example, Serner et al. [27] found 33 different diagnoses used in 72 studies, where most of the diagnostic criteria either were not reported or used in excess. Increasing interest in this specific area has resulted in recent consensus concerning standardisation of taxonomy and terminology [13, 35, 36].

Epidemiological research in football is often described either during one season [2, 18], part of a season [3, 9], or comprised of rather small sample sizes [22]. Extensive research has been conducted in Europe and Scandinavia at club level, by focusing on injury incidence and injury pattern [32]. However, there are fewer studies concentrating on hip/groin injuries, where the focus has been on a selection of European teams [37, 38] or non-professionals [20, 31]. Nonetheless, there was one recent study that used injury surveillance to describe hip/groin injuries in a professional league [23]. Despite the growing amount of hip/groin research in football, little is known about seasonal variations in professional league football. To the best of the author’s knowledge, no previous study has investigated variations between seasons for an entire professional league.

The aim of the present cohort study was to investigate the incidence, pattern, and burden of hip/groin injuries in Swedish professional male football players over five consecutive seasons.

## Materials and methods

The present cohort study of Swedish professional male football players was carried out over five consecutive seasons, from 2012 to 2016. The Swedish first football league consists of 16 teams annually. Over five seasons, there has been a total of 80 team seasons (2,299 players, age range 16–44), with 23 different teams competing. Four of these teams, equal to 400 players and 14 team seasons, have not collected data for the Swedish National Injury Surveillance Database [30] on annual basis and are, therefore, excluded in the present study. One team, equal to 154 players and five team seasons, did not submit their written consent in time for data extraction and is, consequently, excluded from the present study. Two teams, equivalent to 58 players and two team seasons, registered data between the 2012 and 2015 season and are accordingly included in those seasons in the present study.

Ethical approval was obtained from the Regional Ethical Review Board in Umeå, Sweden (2016/491-31), and the study was conducted in accordance with the Declaration of Helsinki for Human studies.

### Data collection

A total of 59 team seasons were included, involving 16 different teams, with seven of the teams participating during all five seasons. Teams participated in a mean±SD of 3.7±1.5 seasons (range 1–5). There were 1,687 players in total, with an average of 29 players per team (range 23–34), divided by 11% goalkeepers (*n* = 186), 35% defenders (*n* = 585), 34% midfielders (*n* = 579), and 20% forwards (*n* = 337). Anthropometric data for the included football players were 25±5 years (range 16–44), with a height of 183±6 cm (range 166–200) and weight of 78±7 kg (range 53–96).

Staff members from all 16 medical teams were initially contacted, between December 2016 and February 2017, and provided with oral and written information about the study.

All teams consented in writing to participate in the present study. Data regarding player and team characteristics (player position, age, height, weight and squad size) were collected through the website Elitefootball.com [10], which provides such data on the official website of the Swedish first football league [24]. All other data, such as total team exposure (training and playing time), injuries (time-loss), and absence days (days lost due to injury), were extracted from the Swedish National Injury Surveillance Database [30].

This injury surveillance database is used prospectively by medical teams in the Swedish first football league to report injuries and treatment of individual players. Injuries that cause time-loss are registered with an injury card, which follows the consensus on injury definitions and data collection procedures in football studies [11]. This reporting system enables extraction of data for statistical purposes and to design epidemiological studies on football injuries.

### Definitions

All injuries sustained by a player during football play (training and/or match) and resulting in time-loss (a player being unable to participate fully in future training or match play) are defined as injury and should be registered in the Swedish National Injury Surveillance Database [30]. A recurrent injury is defined as an injury occurring after a player returns to full participation from an index injury of the same type and at the same site as the first injury [11]. In accordance with the consensus on injury definitions and data collection procedures in football studies, injury incidence is calculated as the number of injuries per 1000 exposure hours [11]. Injury burden is calculated as the number of days lost per 1000 h of exposure, according to Bahr et al. [5].

### Statistical analysis

Collected data were analysed using Microsoft Excel 2016 ver. 15.33 (Microsoft Co. Redmond, WA, USA) and IBM SPSS Statistics ver. 24.0 (IBM Co. Armonk, NY, USA). Descriptive statistics are presented as frequency, percent, mean, standard deviation (SD), 95% confidence interval (95% CI), median, interquartile range (IQR), and range. Inferential statistics were used when analysing incidence and burden between seasons. A one-way analysis of variance (ANOVA) was used to compare normally distributed data, and the Kruskal–Wallis *H* test was used when data were not normally distributed. The significance level was set at 5% (*p* < 0.05).

## Results

In total, 467 time-loss injuries located to the hip/groin were recorded among 1687 professional football players. The total exposure (training and matches) for 59 team seasons was 566,145 h, with a mean ± SD of 9596 ± 2362 h per season. As a result, injury incidence was 0.82/1000 h (95% CI 0.71–1.01). Results did not reveal any significant differences between seasons *F*(4, 54) = 1.42, n.s. The median prevalence of time-loss injuries was 7.0 (IQR 4.5–11.0) per season per club, with an average of 29 players.

The overall injury burden was 15.6/1000 h (95% CI 11.9–20.6). Results showed that there was no statistically significant difference in injury burden between seasons, *X*^2^(4) = 5.401, n.s., with a mean rank burden of 30.58 for the 2012 season, 37.30 for the 2013 season, 21.50 for the 2014 season, 33.38 for the 2015 season, and 28.17 for the 2016 season. The median day lost due to injury was 97 days (IQR 50–209) per season per club. Descriptive data regarding frequency, incidence, and burden are summarised in Table 1.

**Table 1:** Frequency of hip/groin injuries in Swedish professional football

Season	2012	2013	2014	2015	2016	Total
No. of injuries	94	76	78	118	101	467
No. of training injuries (%)	66 (70%)	53 (70%)	53 (68%)	79 (67%)	67 (66%)	318 (68%)
No. of match injuries (%)	28 (30%)	23 (30%)	25 (32%)	39 (33%)	34 (34%)	149 (32%)
No. of injuries per club^a^	6.5 (3.0–11.3)	7.0 (4.5–10.5)	5.0 (2.0–9.3)	7.0 (6.0–10.0)	8.0 (5.0–11.0)	7.0 (4.5–11.0)
No. of absence days	1889	2147	1146	2427	1234	8843
No. of training absence days (%)	1412 (75%)	1657 (77%)	914 (80%)	1977 (81%)	725 (59%)	6685 (76%)
No. of match absence days (%)	463 (25%)	490 (23%)	232 (20%)	360 (15%)	509 (41%)	2054 (23%)
No. of absence days per club^a^	155 (56–225)	209 (146–243)	59 (26–107)	109 (59–172)	68 (48–133)	97 (50–209)
No. of exposure hours	127 571	110 776	118 759	112 741	96 299	566 145
Incidence^b^	0.74 (0.41–1.05)	0.69 (0.39–1.02)	0.66 (0.33–1.07)	1.05 (0.65–1.51)	1.05 (0.71–1.39)	0.82 (0.71–1.01)
Injury burden^c^	14.8 (8.0–20.5)	19.4 (10.7–29.6)	9.6 (2.4–19.1)	21.5 (6.3–39.0)	12.8 (5.8–21.7)	15.6 (11.9–20.6)

^a^Data presented as median (IQR)

^b^Incidence presented as injuries/1000 h of exposure (95% CI)

^c^Injury burden presented as absence days/1000 h of exposure (95% CI)

Characteristics of hip/groin injuries are presented in Table 2, and types of injuries in hip/groin are presented in Table 3.

**Table 2:** Characteristics of hip/groin injuries

	Number	Percentage	Incidence/burden
Injuries			
Overuse	258	55	0.46^a^
Traumatic	209	45	0.37^a^
Absence days			
Overuse	6022	69	10.6^b^
Acute/trauma	2719	31	4.8^b^
Injuries			
Index injuries	402	86	0.71^a^
Recurrent injuries	65	14	0.11^a^
Absence days			
Index injuries	7239	83	12.8^b^
Recurrent injuries	1500	17	2.6^b^

Percentage refers to the total number of injuries and absence days in this table

^a^Incidence and ^b^burden are presented as injuries and absence days/1000 h of exposure respectively

**Table 3:** Injury type of hip/groin injuries

	Injuries *n* (%)	Incidence	Absence days *n* (%)	Burden
Muscle rupture/tear/strain/cramps	278 (60)	0.5	3292 (39)	6.0
Unspecific overuse symptoms	113 (24)	0.2	3123 (37)	5.7
Tendon injury/rupture/tendinosis/bursitis	28 (6)	0.05	419 (5)	0.7
Other injuries	23 (5)	0.04	1407 (16)	2.5
Contusion	17 (4)	0.03	117 (1)	0.2
Haematoma	4 (1)	0.007	79 (1)	0.1
Synovitis/joint swelling	4 (1)	0.007	75 (1)	0.1

Percentage refers to the total number of injuries and absence days in this table

Incidence are presented as injuries/1000 h of exposure. Burden are presented as absence days/1000 h of exposure

**Fig. 1:**
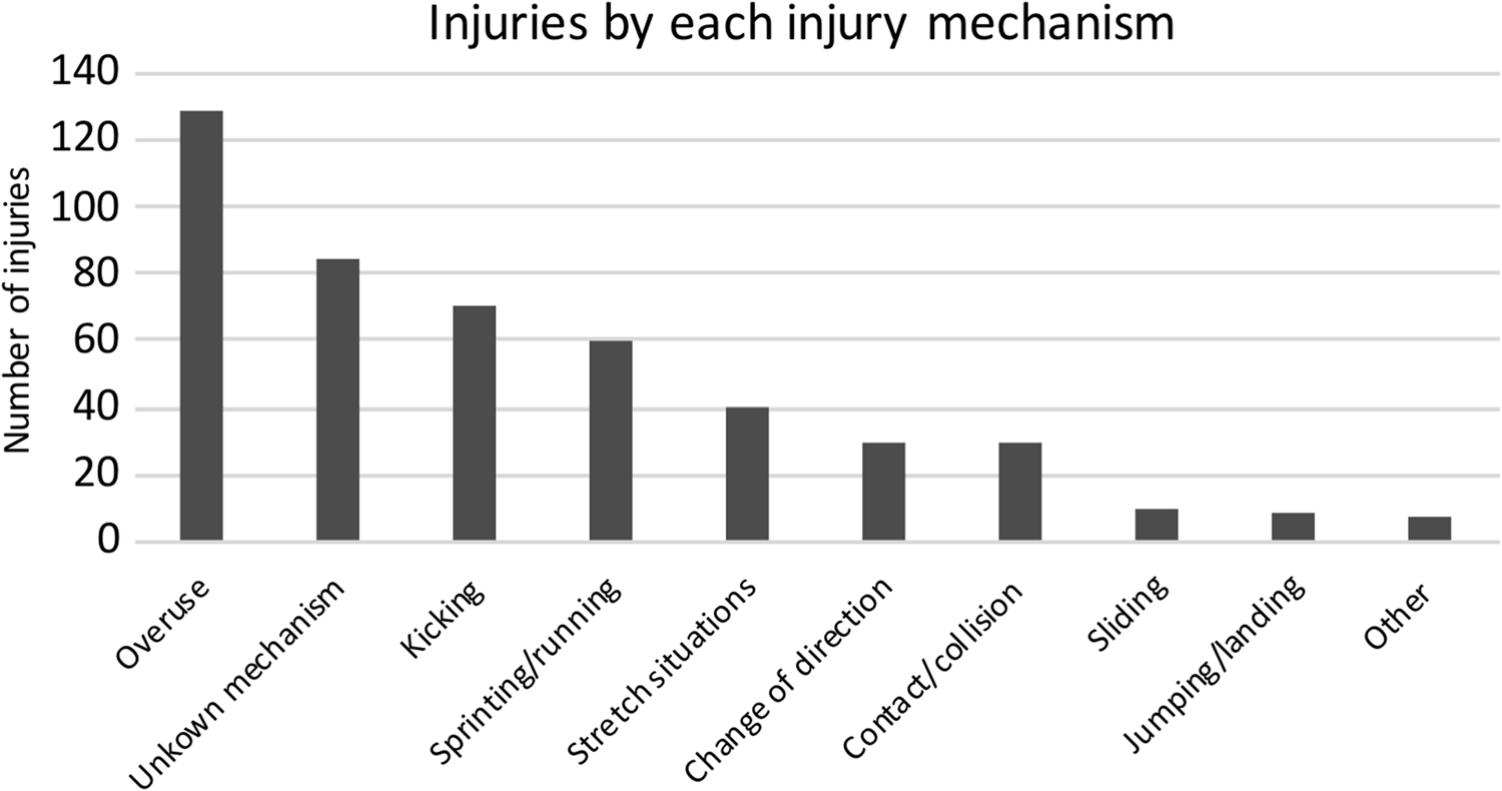
Total number of injuries by each injury mechanism during the 2012–2016 seasons

**Fig. 2:**
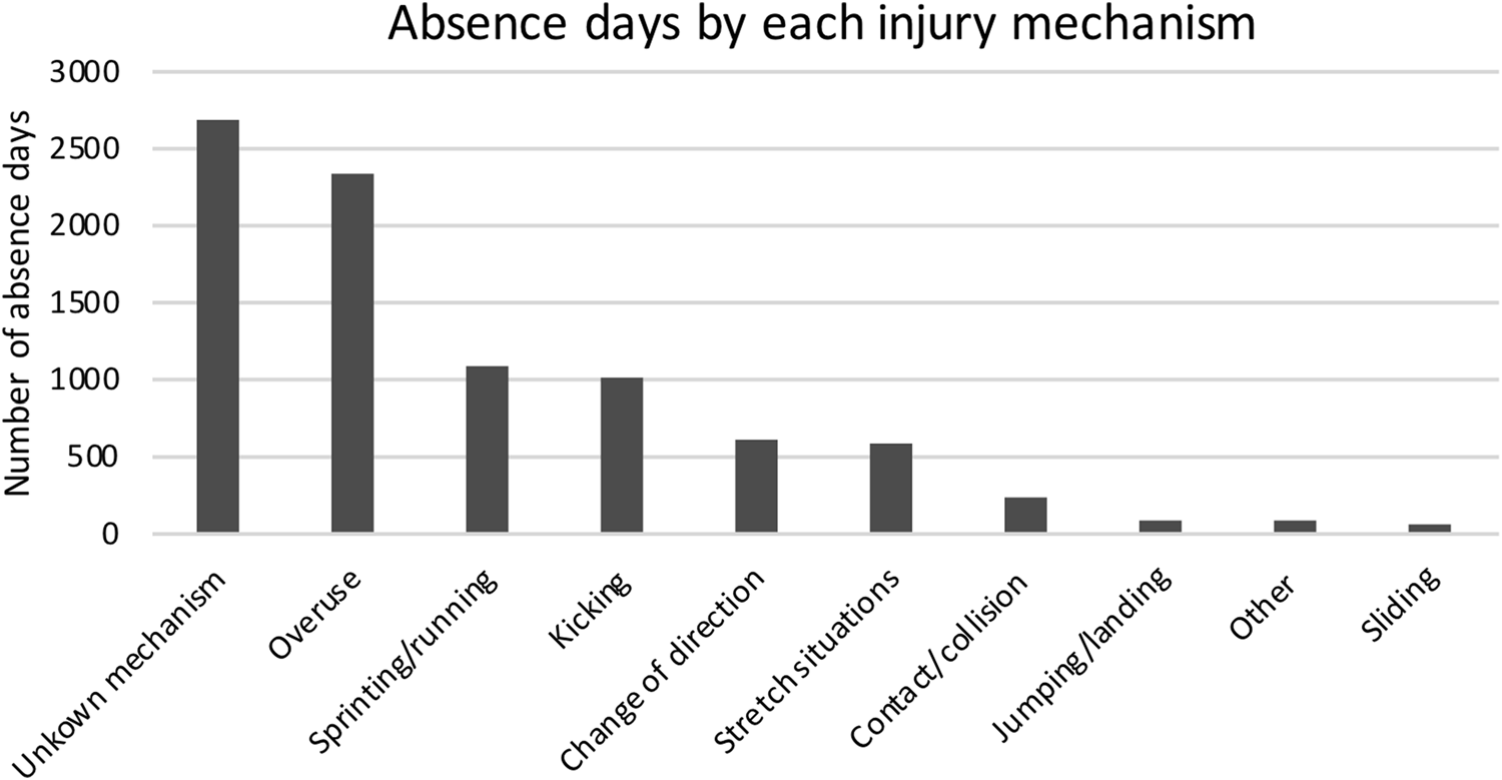
Total number of absence days by each injury mechanism during the 2012–2016 seasons

Overuse mechanism was found to be the most frequent determining factor, with 28% injuries, followed by unknown mechanism (18%), kicking (15%), sprinting/running (13%), and stretch situations (9%) (Fig. 1). However, when looking at absence days from football play, the unknown mechanism was the most commonly observed (31%), followed by overuse (27%), sprinting/running (12%), kicking (11%), change of direction (7%), and stretch situations (7%) (Fig. 2).

### Player position

Defenders sustained most hip/groin injuries, which also were the most severe ones, followed by midfielders, forwards, and goalkeepers (Table 4). However, when adjusting for the number of players in their respective positions, both defenders and midfielders sustained 29% each of the total injuries, but defenders alone sustained most of the absence days (33%) (Table 4).

**Table 4:** Injuries and absence days by position

	Injuries	Percentage^a^	Absence days	Percentage^a^
Goalkeeper	27	6	390	5
Defender	165	38	3387	41
Midfielder	160	36	2723	33
Forward	88	20	1682	21

^a^Percentage refers to the number of injuries and absence days per position divided by the total number of injuries and absence days in this table

## Discussion

The main finding in the present cohort study was that hip/groin injuries do not seem to be an increasing phenomenon in the Swedish male first football league. In addition, index and overuse injuries accounted for the majority of injuries and absence days. Therefore, more attention should be paid to preventing hip/groin injuries to lower the injury rate.

### Hip and groin injury epidemiology

The total incidence of hip/groin injuries was 0.82/1000 h, which is lower than the previous research from professional football in Sweden (range 1.0–1.3/1000 h) [17, 18] and the reported rate (1.0/1000 h) in the UEFA Elite Club Injury Study [37]. The incidence was, however, greater than those reported from Scandinavian sub-elite and amateur level [2, 9, 20], which might suggest that hip/groin injuries tend to increase with higher level of play. One explanation for this trend could be the heavier training and match load for the most successful teams in Europe, which play both domestic and international games such as in the UEFA Champions League and Europa League. Players from the best teams also tend to play for their national teams, which further increases their seasonal workload. Other factors that hypothetically might influence injury risk could be differences in tactics, climate, field surface, playing style, and match intensity. In addition, Waldén et al. [34] concluded that regional differences in injury epidemiology exist and should be considered when comparing football studies. However, there was a trend towards an increasing incidence over five consecutive seasons in the present study, with the injury rate being approximately 43% higher during the last two seasons (2015–2016) than the initial three (2012–2014). However, this comparison did not reach statistical significance. This is an interesting finding, since it differs from the reported data in the UEFA Elite Club Injury Study, where the incidence was found to be slightly decreasing over a 15-year period of time [37]. One potential reason for this sudden increase in incidence during the 2015 season could partly be explained by the publication of the consensus statement on hip/groin injury definitions and terminology the same year [35]. With a better understanding about diagnostic criteria, medical teams might have captured more injuries leading to an increase in incidence.

Nevertheless, when comparing seasonal incidence in the present study with the previous studies from professional male football in Sweden, the injury rate lies around 1.0 injury per 1000 exposure hours (range 0.7–1.3, median 1.0) [17, 18]. This suggests that the injury incidence is not an increasing phenomenon in Swedish professional male football over a longer period of time, and that seasonal changes happen more often due to natural variations. This is somewhat supported by Bjørneboe et al. [6] who found acute hip/groin match injury incidence to be fluctuating over six consecutive seasons in the Norwegian professional league, with approximately the same range (0.6–1.4) as earlier studies, including the present one, have reported [17, 18].

The seasonal burden was found to be more inconsistent (range 9.6–21.5 absence days) than the injury rate, and the present study could not present any increasing trend. Burden refers to the risk of absence from football play, which could vary depending on the severity of injuries. Like incidence, burden can also mean natural variations between seasons that hypothetically can depend on factors such as coaching philosophy, seasonal workload, and injury management.

Burden has not been a standard measure in earlier studies; therefore, there is limited research for comparisons. Thus, the overall injury burden of hip/groin injuries was 15.6/1000 h and is lower than the 24.3/1000 h found in Qatar’s first league [23]. This difference could partly depend on the number of seasons investigated, where Mosler et al. [23] only studied two seasons compared to five in the present one. However, new data from Werner et al. [37] suggest that the injury burden in the Swedish first football league is almost the same as in the UEFA Elite Club Injury Study, where the rate is 16.1/1000 h.

Recently published time-trend analysis of ankle and hamstring injuries in the UEFA Champions League makes it possible to compare injury risk with the hip/groin findings in the present study. The overall incidence and burden are slightly higher for both ankle (1.0/1000 h and 16.3/1000 h) [33] and hamstring injuries (1.2/1000 h and 19.7/1000 h) [8] compared to the results of hip/groin injuries (0.8/1000 h and 15.6/1000 h) in the present study. However, when comparing UEFA Champions League incidence between the different injury locations, injury rate is surprisingly consistent (hip/groin 1.0/1000 h, ankle 1.0/1000 h, hamstrings 1.2/1000 h) [8, 33, 37], establishing hip/groin injuries as a substantial problem in professional football.

A considerably large amount of hip/groin injuries appear every season in Swedish professional football, and the true magnitude might be underestimated, because the current methodology only covers time-loss injuries [14]. Since football injuries seem to be the most common cause for players being unavailable for football activities during the competitive season [25] and can affect team performance negatively [19], teams are recommended to implement evidence-based preventive training exercises. One potential exercise that teams could consider is the Copenhagen hip adduction exercise due to its eccentric strengthening of the adductors [15, 21]. Teams should also be aware of the fluctuations in both incidence and burden, since these measures affect player availability and thereby team success [19].

### Injury characteristics

The present study displayed a relatively low recurrence rate for hip/groin injuries (14%) compared to the previous findings [1, 16, 23, 38], where injury rates have been found to vary between 15 to 50%. The higher rates were primarily found in studies comprising rather small sample sizes or few seasons, which could partly explain the differences. Nonetheless, recently published data from the UEFA Elite Club Injury Study suggest that the recurrence rate is even lower (11%) [37]. This may be related to greater investments in medical teams in Europe’s top division teams that hypothetically would result in better care of players than at lower levels.

One can argue that recurrent injuries are a medical team issue, where players get a suboptimal rehabilitation and are forced into participating in football activities earlier than required.

The majority of hip/groin injuries in the present study are, however, a result of overuse and primarily affected muscles, as found in the other studies [20, 23, 38]. Overuse injuries are strongly related to drastic changes (spikes) in weekly workload and have lately been considered as a training load error [12], which could suggest that the seasonal workload is the main problem concerning hip/groin injuries in the Swedish first football league and needs to be managed better.

The findings in the present study of a high prevalence of unknown mechanism and overuse mechanism could, to some degree, be explained by the previous lack of consensus concerning terminology and taxonomy [35, 36]. Implementation of these recommendations is strongly recommended to ensure correct diagnosis. Another issue could be the injury registration. If an injury appears during training and the medical staff are not around at the exact moment and the player cannot remember how the injury happened, it could end up as an unspecific or overuse mechanism during injury registration.

As for the specific mechanisms, kicking was the main cause of injury, while sprinting/running represents most of the absence days. These two risk factors have recently been associated with the main mechanisms in rectus femoris injuries [29], whereas kicking and change of direction seem to be the main risk factors for adductor longus strains [28]. Together with stretch situations, these four injury situations confirm the previous research as the key mechanisms in team sport athletes [26]. Further conclusions could not be stated in the present study and should be addressed in future studies.

The main strength of the present study is that the data collections were conducted prospectively over several seasons and include a substantial homogenous group of professional football players. In addition, data were obtained from an injury surveillance database, which is developed for statistical purposes, and follows the international consensus agreements on injury definitions and data collection procedures in epidemiological studies on football injuries [11]. Another strength is the implementation of injury burden that together with incidence illustrates a relationship between the consequence (burden) and the likelihood (incidence) of injuries [5]. Since the present study is only the third one to use injury burden as a measure of absence risk for hip/groin injuries in professional football, it could be used in the future to compare burden in football studies.

A clear limitation of the present study is the use of a time-loss injury definition that may underestimate the risk of overuse injuries [4, 14], which represents at least 50% of hip/groin injuries [20, 23, 37, 38]. Therefore, a combined method that captures “all physical complaints” is recommended, but, since the present study is epidemiological, it cannot demonstrate the cause-and-relationship effect. In addition, the present study does not present the overall injury rate, which would have been beneficial to conclude the extent of hip/groin injuries in the Swedish first football league. In addition, it would have been a strength if the present study reported hip and groin injuries separately, as these could be defined as two different anatomical areas. However, when combined, it makes it easier to compare with earlier studies, because this definition is still a consensus [11]. Furthermore, the present study does not report specific diagnosis or clinical entities, which would have been preferable, since a few studies have addressed this topic [32]. Finally, the results in the present study could potentially have been affected by missing data and inaccuracies in the data set. However, injury registration is done by professional medical teams and substantial guidelines are available, both within the injury surveillance database and through evidentially consensus recommendations [11].

## Conclusions

The present study suggests that hip/groin injuries are a substantial problem in football, but does not seem to be an increasing phenomenon in the Swedish male first football league. Index and overuse injuries accounted for the majority of injuries and absence days. Risk factors include mechanisms such as kicking, sprinting/running, change of direction, and stretch situations, and being an outfield player. Medical and fitness staff should focus on strategies to prevent first-time injuries and overuse symptoms transforming into injuries to decrease the hip/groin injury rate.
